# Long-term variations in PM_2.5_ concentrations under changing meteorological conditions in Taiwan

**DOI:** 10.1038/s41598-019-43104-x

**Published:** 2019-04-29

**Authors:** Fang-Yi Cheng, Chia-Hua Hsu

**Affiliations:** Department of Atmospheric Sciences, National Central University, Taoyuan, Taiwan

**Keywords:** Atmospheric dynamics, Environmental impact

## Abstract

With emission control efforts, the PM_2.5_ concentrations and PM_2.5_ exceedance days (daily mean PM_2.5_ concentrations >35 µg m^−3^) show an apparent declining trend from 2006–2017. The PM_2.5_ concentrations increase from the northern to southern part of western Taiwan, and reductions in the PM_2.5_ concentration generally decrease from northern to southern part of western Taiwan. Thus, mitigation of the PM_2.5_ problem is less effective in southwestern Taiwan than in other regions in Taiwan. Analysis of a 39-year ERA-interim reanalysis dataset (1979–2017) reveals a weakening of the East Asian winter monsoon, a reduction in northeasterly (NE) monsoonal flow, and a tendency of enhanced stably stratified atmospheric structures in Taiwan and the surrounding area. The observed surface wind speed also presents a long-term decline. We can conclude that the long-term PM_2.5_ variations in Taiwan are mainly associated with changes in local anthropogenic emissions and modulated by short-term yearly variations due to strong haze events in China. In southwestern Taiwan, the long-term trend of PM_2.5_ reductions is possibly offset by worsening weather conditions, as this region is situated on the leeside of the mountains and often subject to stagnant wind when under the influence of NE monsoonal flow.

## Introduction

Fine particulate matter (PM_2.5_) pollution has become a significant public concern in Taiwan in recent years, particularly regarding the occurrence of severe haze events. Domestic anthropogenic emissions primarily originate from urban areas, coal-fired power plants, oil refinery plants, industrial parks and major highways mostly in western Taiwan^1^. To improve air quality, Taiwan’s government has applied numerous emissions control strategies, such as a gasoline vapor recovery program that has been advocated for gas stations since 1997, air pollution control devices that have been employed to reduce fugitive emissions of highly reactive volatile organic compounds and hazardous air pollutants from petrochemical and refining industries since 1998, control of fugitive particulate emissions from stationary sources since 2009, and strict vehicle emission standards and on-road vehicle emission control measures^1–3^. Despite these emission control measures, severe PM_2.5_ events continue to occur frequently in Taiwan^4^.

**Figure 1:**
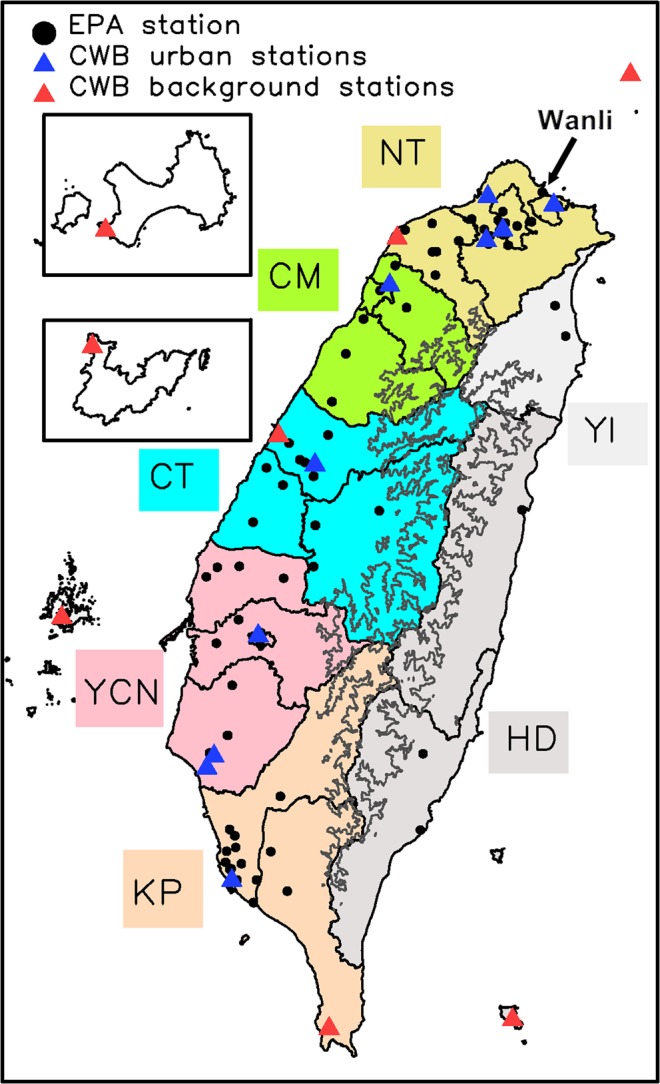
Location of the surface stations. Black points indicate the EPA surface stations. Blue and red triangles indicate the CWB surface stations. Red triangles indicate the CWB background stations. The seven AQZs (NT, CM, CT, YCN, KP, and YI and HD) are identified by name. The contour line indicates the mountain range (CMR).

According to geographical and meteorological conditions and the nature of air contaminants, the Taiwan Environmental Protection Administration (TEPA) has divided the nation into seven air quality zones (AQZs), namely, northern Taiwan (NT), the Chu-Miao (CM) area, central Taiwan (CT), the Yun-Chia-Nan (YCN) area, the Kao-Ping (KP) area, the Hua-Dong (HD) area, and Yilan (YI) (Fig. 1). Within the AQZs, we can assume that air pollution behavior is less distinct. Among the seven AQZs, KP faces the most serious air pollution problems, which are associated with emissions as well as meteorological conditions^5–10^. From autumn to the following spring season, the PM_2.5_ concentration frequently violates the national standard due to the reduced atmospheric ventilation capability^11^. Hsu and Cheng^4^ indicated that the occurrence of PM_2.5_ episodes in Taiwan is typically associated with unfavorable meteorological conditions. Taiwan is an island characterized by a central mountain range (CMR, with peaks as high as 3952 m) that spans from the north to the south of the island and is flanked by gently sloping plains to the west. When the prevailing northeasterly (NE) to easterly wind (depending on the location of the Asian continental anticyclone system) is obstructed by the CMR, low wind speeds and strong subsidence occur over the leeside of the mountains (CT/YCN/KP areas), which often leads to serious PM_2.5_ accumulation and causes severe PM_2.5_ events; the most serious PM_2.5_ problem is in the KP area.

Several previous studies have indicated that changes in climate and global circulation can affect air pollution dispersion^12–19^. Kim *et al*.^20^ showed that emission control efforts by the South Korean government and neighboring countries seem to be effective in reducing PM_2.5_ concentrations; however, changes in meteorological conditions due to interannual variability in regional wind speed seem to offset these efforts. Studies have examined the climate change characteristics in Taiwan and found a warming tendency that was closely related to large-scale circulation changes, including the weakening of East Asian monsoons^21,22^.

Given the strong connection between the weather conditions and PM_2.5_ concentrations, in the following, we examined the long-term variations in PM_2.5_ concentrations in Taiwan and meteorological conditions in East Asia and Taiwan and studied the implications of meteorological variations on surface PM_2.5_ concentrations in Taiwan. Studies conducted in Taiwan have examined the long-term trends of air pollutants^23–26^; however, none of these studies discussed the impact of meteorological variations under regional climate change on the long-term PM_2.5_ concentrations.

## Results

### Long-term variation in the NO_x_, SO_2_ and PM_2.5_ concentrations

**Figure 2:**
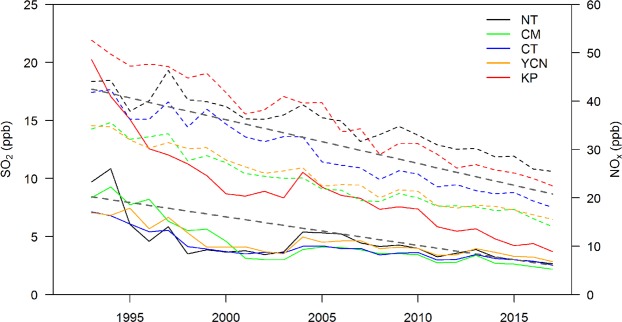
Variations in the average NO_x_ (dashed lines) and SO_2_ (solid lines) concentrations for the five AQZs over 1993–2017. The trend line is given for the area mean NO_x_ and SO_2_ concentrations.

**Figure 3:**
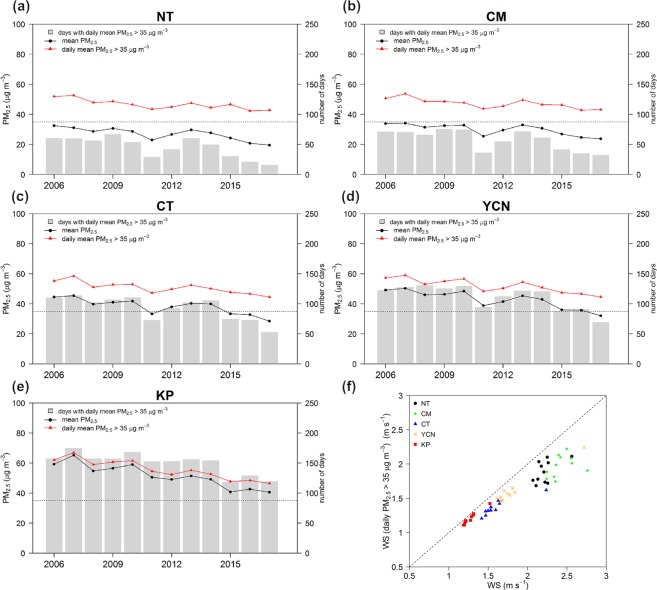
(**a**–**e**) Long-term variations in PM_2.5_ concentration. The horizontal black dashed line identifies the PM_2.5_ concentration at 35 μg m^−3^, black lines denote the mean PM_2.5_ concentration, and red lines denote the daily mean PM_2.5_ concentration >35 μg m^−3^. Gray bars indicate the number of days with a daily mean PM_2.5_ concentration >35 μg m^−3^. (**f**) Annual wind speed comparison between the mean wind speed and the wind speed associated with daily mean PM_2.5_ concentrations >35 μg m^−3^.

The average NO_x_ and SO_2_ concentrations from 1993–2017 for the five AQZs (NT/CM/CT/YCN/KP) in western Taiwan are presented in Fig. 2. The average PM_2.5_ concentrations from 2006–2017 are presented in Fig. 3. The PM_2.5_ analysis starts in 2006 because most PM_2.5_ observations in Taiwan were established in 2006. The HD and YI AQZs are not discussed due to the relatively low impact of air pollution on these areas. This study mainly focuses on addressing the air pollution problem; therefore, the analysis only considers the six-month air pollution season (October to March). The long-term trend of the average NO_x_ and SO_2_ concentrations clearly declined from 1993–2017. The highest reduction in NO_x_ and SO_2_ concentrations occurred in the KP area. The variations in SO_2_ concentrations have been quite stable in most AQZs since 1998, except in the KP area. Compared to the mean NO_x_, SO_2_ and PM_2.5_ concentrations in 2006, those in 2017 were reduced by 32%, 49% and 35%, respectively, because of the emission control efforts.

According to the air quality standard of TEPA, a day on which the daily mean PM_2.5_ concentration >35 µg m^−3^ is considered a PM_2.5_ exceedance day. Figure 3 also reveals the annual variations in the average PM_2.5_ concentrations on the exceedance days and the number of PM_2.5_ exceedance days. Moreover, an annual wind speed comparison between the mean wind speed and the wind speed on PM_2.5_ exceedance days using data from TEPA surface air quality monitoring stations is presented in Fig. 3f.

The long-term trend analysis indicates gradual declines in the PM_2.5_ concentration and the occurrence frequency of PM_2.5_ exceedance days. The PM_2.5_ concentrations and the occurrence frequency of PM_2.5_ exceedance days increase from the northern to southern part of western Taiwan. The highest PM_2.5_ concentration occurs in the KP area followed by the YCN and CT areas, while the lowest wind speed occurs in the KP area followed by the CT and YCN areas (Fig. 3f). The wind speed on PM_2.5_ exceedance days is lower than the mean wind speed overall. In general, the PM_2.5_ exceedance days are associated with stagnant wind conditions in the CT/YCN/KP areas. Over the NT/CM areas, the wind speed is higher and the variability in the wind speed on the PM_2.5_ exceedance days is larger than those over the CT/YCN/KP areas. Under the influence of the Asian continental anticyclone system, NE monsoonal flow can transport air pollution from China into Taiwan, and NT/CM are more vulnerable to transboundary air pollution than the CT/YCN/KP areas^4^. High PM_2.5_ concentrations in the NT/CM areas can be increased by transboundary air pollution under the influence of strong NE wind; however, low wind speed can accumulate local air pollution and cause PM_2.5_ events, which explains the large variability in the wind speed on the PM_2.5_ exceedance days in the NT/CM areas.

Since 2006, there has been an apparent reduction in the PM_2.5_ concentrations and PM_2.5_ exceedance days in the five AQZs. Mitigation of the PM_2.5_ problem is most apparent in the NT area, where the mean PM_2.5_ concentration was reduced from 32.45 µg m^−3^ in 2006 to 19.53 µg m^−3^ in 2017, and the PM_2.5_ exceedance days were reduced from 60 days in 2006 to only 15 days in 2017. In southwestern Taiwan, the KP area, which often exhibits stagnant wind fields, had a mean PM_2.5_ concentration of 40.61 µg m^−3^ in 2017, exceeding the standard, and 119 days (approximately 67% of the total sampling days) violated the daily PM_2.5_ standard. In particular, during the most recent three-year period (2015–2017), the reduction in the PM_2.5_ concentrations and the PM_2.5_ exceedance days was very small in the KP area.

Furthermore, we estimated the variation ratio of PM_2.5_ concentrations (PM_2.5_ exceedance days) in each year relative to that in the previous year. The annual variation ratio of the PM_2.5_ concentrations (PM_2.5_ exceedance days) for the five AQZs averaged from 2006 to 2017 is presented in Table 1. In general, the reduction in the PM_2.5_ concentrations (PM_2.5_ exceedance days) decreases from the northern to southern parts of western Taiwan. The annual variation ratios of PM_2.5_ concentrations and PM_2.5_ exceedance days are −3.94% and −7.17%, respectively, in the NT area, while they are −2.96% and −1.98%, respectively, in the KP area. Thus, mitigation of the PM_2.5_ problem is less effective in the KP area than in the other AQZs.

**Table 1:** Annual variation ratio of the PM_2.5_ concentrations and the PM_2.5_ exceedance days for the five studied AQZs averaged from 2006 to 2017.

AQZ	NT	CM	CT	YCN	KP
Ratio of PM_2.5_ concentrations (%)	−3.94	−2.59	−3.42	−3.36	−2.96
Ratio of PM_2.5_ exceedance days (%)	−7.17	−3.05	−4.67	−3.87	−1.98

Since the occurrence of high PM_2.5_ concentrations is typically associated with unfavorable meteorological conditions in Taiwan^4^, there is a possibility that the long-term variations in meteorological conditions can affect the variations in the PM_2.5_ concentrations.

### Meteorological conditions in East Asia and Taiwan (1979–2017)

The long-term analysis by the Central Weather Bureau (CWB) surface weather stations in Taiwan indicated an increasing trend of surface temperature and a decreasing trend of surface pressure (Fig. S1 in the Supplementary Information) from 1979 to 2017. The locations of the CWB sites are illustrated in Fig. 1. An apparent warming has been observed since 1997 in Taiwan.

**Figure 4:**
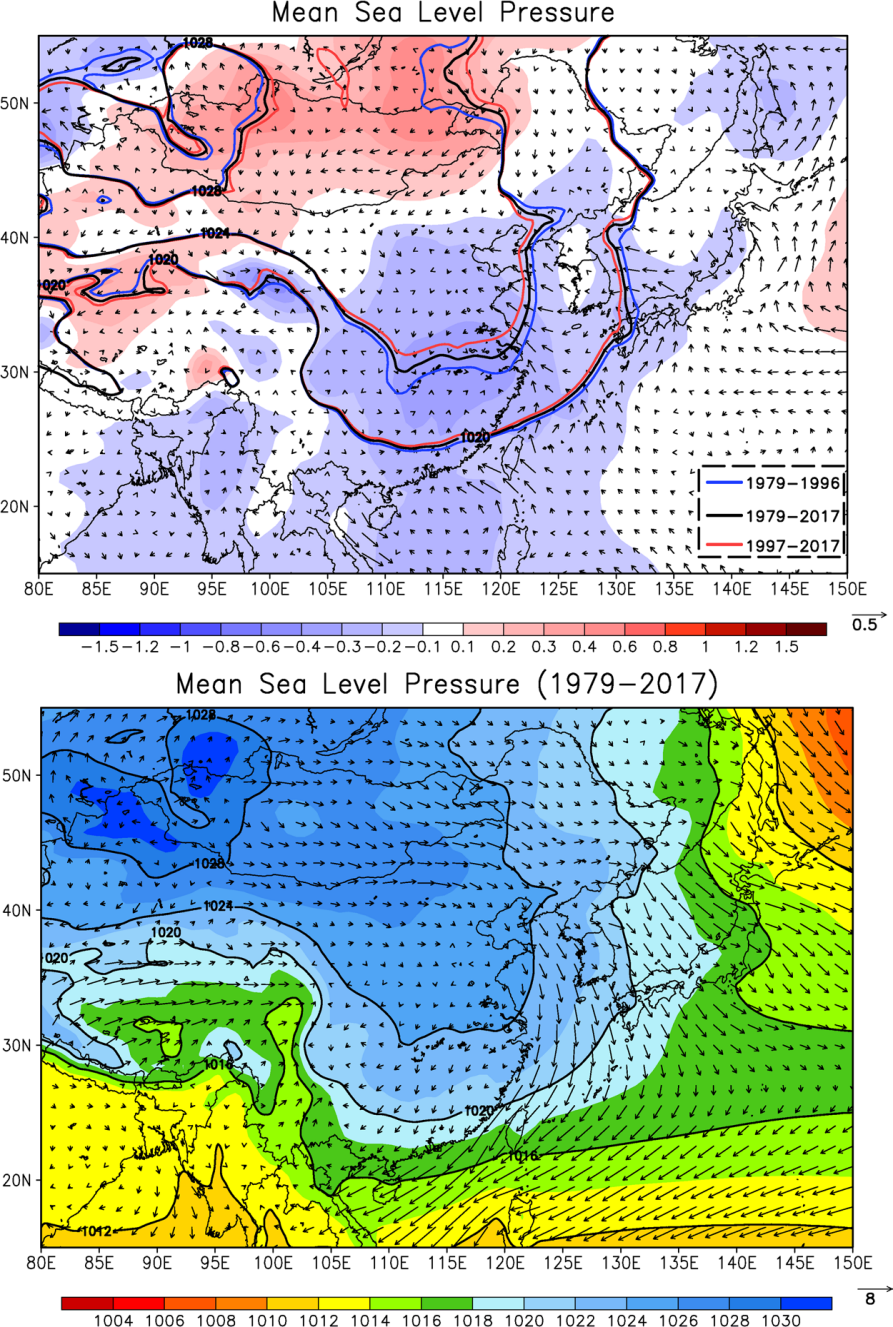
Distribution of the MSLP. In the upper panel, blue, black and red contours denote the mean MSLP from 1979–1996, 1979–2017 and 1997–2017, respectively. Shaded colors and wind vectors represent the anomalies of the MSLP and wind from 1997–2017 relative to the 39-year average. The bottom panel is the MSLP and wind vector averaged over the 39-year period (1979–2017).

An analysis of the mean sea level pressure (MSLP) and the wind vector over East Asia is conducted to examine the variations in the East Asian winter monsoon system based on the 39-year ERA-interim reanalysis dataset^27^ (1979–2017) (Fig. 4) under climate warming. The MSLPs from 1979–1996, 1979–2017, and 1997–2017 are averaged separately to represent the past, the 39-year mean, and the current atmospheric conditions. The past and current periods are selected to represent the cold and warm scenarios, respectively, to investigate the influence of climate warming on regional (East Asia) to local (Taiwan) meteorological conditions. Analysis of the mean wind field indicates that NE monsoonal flow prevails over the East China Sea and the Taiwan area. The northward retreat of the East Asian winter monsoon and the negative anomaly of the MSLP distribution over the low to middle latitudes of East Asia are identified in the current warm period, which indicates the weakening of the East Asian winter monsoon system in the current period, i.e., 1997–2017. A weakened East Asian winter monsoon was also identified in a previous study^28^. Additionally, the wind vector anomaly (Fig. 4) shows a reduction in the northerly wind in the current period, particularly over the East China Sea and the Taiwan area.

### Long-term variation in atmospheric temperature profiler

**Figure 5:**
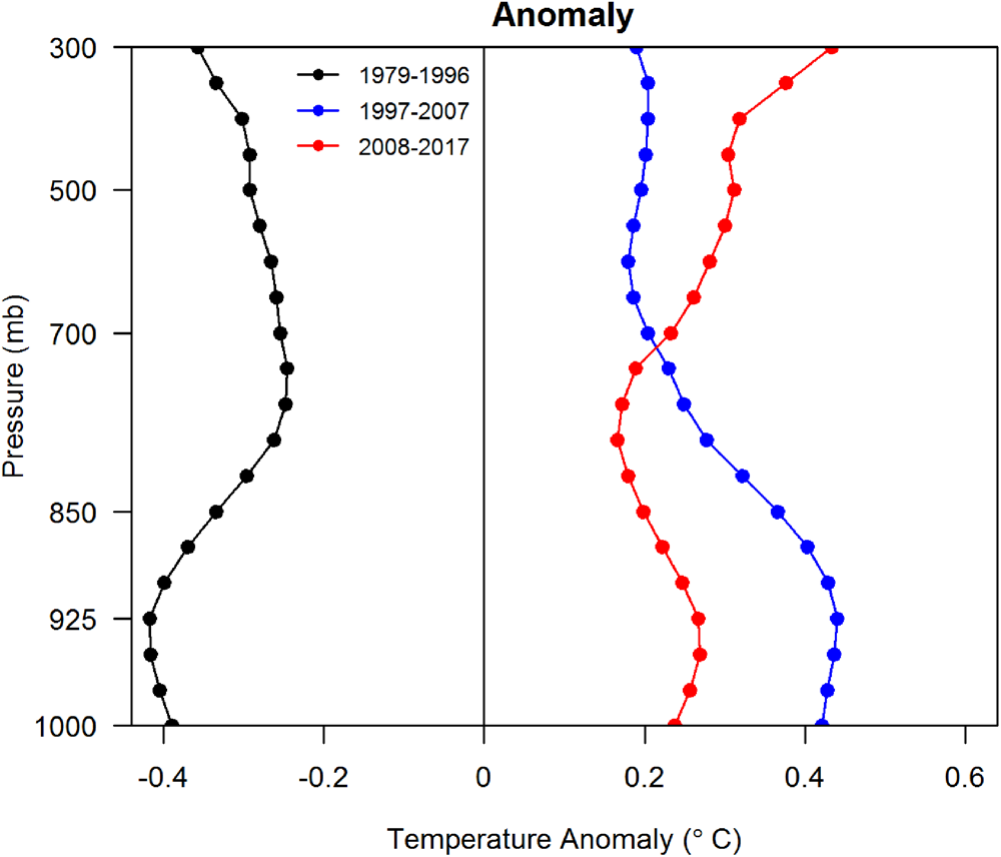
Temperature anomaly vertical profile for 1979–1996 (black), 1997–2007 (blue), and 2008–2017 (red) relative to 1979–2017.

To assess the long-term variations in atmospheric stability, an analysis of the atmospheric temperature profiler was conducted using the 39-year ERA-interim reanalysis dataset. The analysis is conducted over the area surrounding Taiwan (north latitude: 10–40; east longitude: 105–135), where the MSLP has decreased over the current period, as illustrated in Fig. 4. Figure 5 shows the average of the temperature anomaly vertical profile for atmospheric conditions in 1979–1996, 1997–2007, and 2008–2017 relative to the 39-year average (1979–2017). The time period from 1979–1996 represents past conditions (cold scenario). The recent 21-year period (1997–2017) is divided into two segments (1997–2007 and 2008–2017) to explore the detailed variations. Compared to the temperature anomaly of the past period (1979–1996), the positive temperature anomaly of the two recent periods (1997–2007 and 2008–2017) indicates an overall warming structure. In particular, the warming structure is more apparent in the upper layers of the atmosphere (above 700 hPa) than in the bottom layers of the atmosphere over the most recent 10-year period (2008–2017), which indicates a tendency of enhanced atmospheric stability. Additional analysis of the 500-hPa geopotential height reveals a positive anomaly in East Asia in the current warm period (Fig. S2 in the Supplementary Information), which may enhance the upper layer subsidence process and warm the upper air. Upper layer warming has also been reported by Sherwood and Nishant^29^, who analyzed global radiosonde data and observed tropical warming features, with a peak warming near 300 hPa; these authors stated that the increases in warming with height have represented a moist adiabatic profile to 300 hPa since 1959 or 1979. An overall stably stratified atmospheric structure from the surface to the 300-hPa level can have a significant impact on the dispersion of air pollution.

### Wind speed variation in Taiwan (1979–2017)

**Figure 6:**
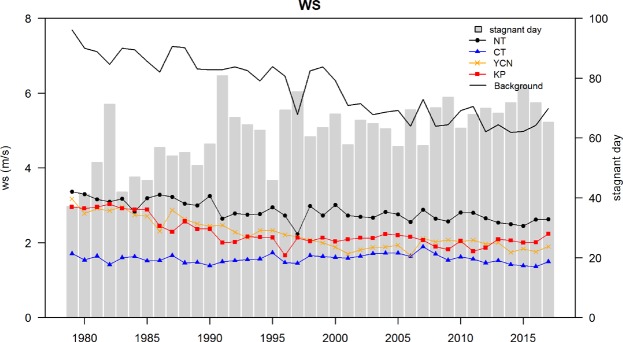
Variation in the wind speed averaged over the six-month air pollution season for different AQZs and the background stations over 39 years (1979 to 2017). The gray bars indicate the number of days with stagnant wind conditions.

Focusing on the area of Taiwan, Fig. 6 shows the variations in observed surface wind speed over the 39-year period (1979–2017) for four AQZs (NT, CT, YCN and KP). The wind speeds are estimated by averaging available data from CWB surface stations located within the individual AQZs. The CM area is not included in the analysis because only one surface station in this zone has available long-term wind speed data; in addition, the data are incomplete for certain years. The wind speed data from the CWB surface weather stations are used instead of the data from the TEPA air quality monitoring stations because CWB has a long period of observations. The averages for the background stations are processed separately to represent the synoptic wind conditions. The background stations consist of the stations located in offshore or coastal areas. The stations used in the individual AQZs are located in urban or suburban areas, and the wind flows are more likely to be affected by local environmental flows, such as land-sea breeze or urban air flow. Moreover, the occurrence frequency of stagnant wind conditions is estimated. A stagnant wind condition day is defined as a day on which the daily mean wind speed averaged from the CWB surface stations is at least 20% lower than the climatological value for the reference period (1979–2017), which follows the National Climatic Data Center methodology^30^. There appears to be a gradual increase in the occurrence of days with stagnant wind conditions.

An apparent wind speed decline is observed in the NT/YCN/KP areas. The wind speed reduction is even more significant for the background sites than for the individual AQZs, which indicates that the wind speed reduction is more likely caused by changes in the synoptic wind than variations in the local land surface. For example, the urban expansion process can increase the surface roughness over the land surfaces and lead to an increase in surface drag and a reduction in surface wind speed^31,32^. From autumn to spring, the prevailing wind in Taiwan is mainly dominated by the NE monsoonal flow. A reduction in the NE wind strongly affects the local air flow in Taiwan. A number of studies have reported declines in near-surface wind speed in China during recent decades^33–35^. Niu *et al*.^36^ found an increase in fog events and a decrease in surface wind speed during winter time over eastern-central China and attributed these changes to the weakening of the East Asian winter monsoon system.

Analysis of the CWB surface wind indicates that among the four AQZs, the wind speed is the lowest in the CT area, where the variation in the wind speed over the past 39 years is less apparent. Please be aware that the observed wind speeds displayed in Figs 3f and 6 are from different observation datasets. In Fig. 3, analysis of the TEPA surface stations indicates that the lowest wind speed is in the KP area; however, the CWB surface station does not reveal this low wind speed in the KP area because there is only one CWB station in the KP area and the station is near a coast that tends to exhibit coastal wind.

### Influence from the transboundary air pollutants

In addition to locally released emissions, the PM_2.5_ concentration in Taiwan can be attributed to transboundary air pollutants from East Asia^8,37,38^, which are mostly observed in winter and spring. For example, long-term analysis of high PM_2.5_ concentrations shows a significant increase in 2013 (Fig. 3), which was attributed to the occurrence of several heavy PM_2.5_ episodes in China; the air pollutants were transported into Taiwan. Wang *et al*.^37^ indicated that six heavy haze events occurred in China during the winter of 2013–2014, which is considered the historically high record.

Studies have indicated that an apparent decreasing trend of PM_2.5_ concentrations has occurred in China^39,40^, and Zheng *et al*.^41^ also reported an apparent declining trend of anthropogenic emissions from China since 2010. We believe that the clean air activity in China can promote reductions in PM_2.5_ concentrations in Taiwan. According to previous studies^37,38,42^, the influence of transboundary air pollution on Taiwan is typically associated with the Asian continental outflow and the NE monsoonal flow. Following those studies, we tried to identify the severe transboundary PM_2.5_ events by using the observed surface wind at the Wanli EPA surface station. Wanli is located on the northeastern coast of Taiwan (Fig. 1); it is approximately 50 m from the shore and has high exposure to transboundary air pollution when under the influence of NE monsoonal flow^42^. Wang *et al*.^37^ used the observed wind data from the Wanli EPA surface station to investigate the transported haze events over northern Taiwan. Junker *et al*.^42^ utilized the Wanli observed wind fields by constraining the wind flow direction and wind speed magnitude to discuss the effect of long-range transport of air pollutants on coastal sites in Taiwan. First, we extracted the times that are dominated by the prevailing NE monsoonal flow based on the observed wind field at the Wanli station. The time period associated with wind flow from the marine sectors (0–75°) is selected; on the other hand, the impact of local air pollution can be minimized because the wind flow is from the ocean and not from Taiwan island. Second, the wind speed has to be higher than 3.6 m s^−1^ to represent NE monsoonal flow. Junker *et al*.^42^ identified a strong negative correlation between wind speed and CO_2_/SO_2_ concentrations for wind speeds less than 3.6 m s^−1^ at Wanli station, which indicates the effect of local pollution. However, when wind speed is higher than 3.6 m s^−1^, CO_2_/SO_2_ tends to stay at similar levels, and this low variation indicates the possible influence of transboundary air pollution through the NE monsoonal flow. With application of the above criteria, the selected time periods can be used to discuss PM_2.5_ variations under the influence of NE monsoonal flow. Furthermore, the selected time periods with hourly PM_2.5_ concentrations >54 µg m^−3^ are used to represent severe PM_2.5_ events due to transboundary air pollution. The selected transboundary PM_2.5_ events match well with the haze events identified by Wang *et al*.^37^. The following discussions are separated into the time periods that are dominated by prevailing NE monsoonal flow (i.e., NEM) and the remaining time periods excluding the NEM periods (i.e., noNEM).

**Figure 7:**
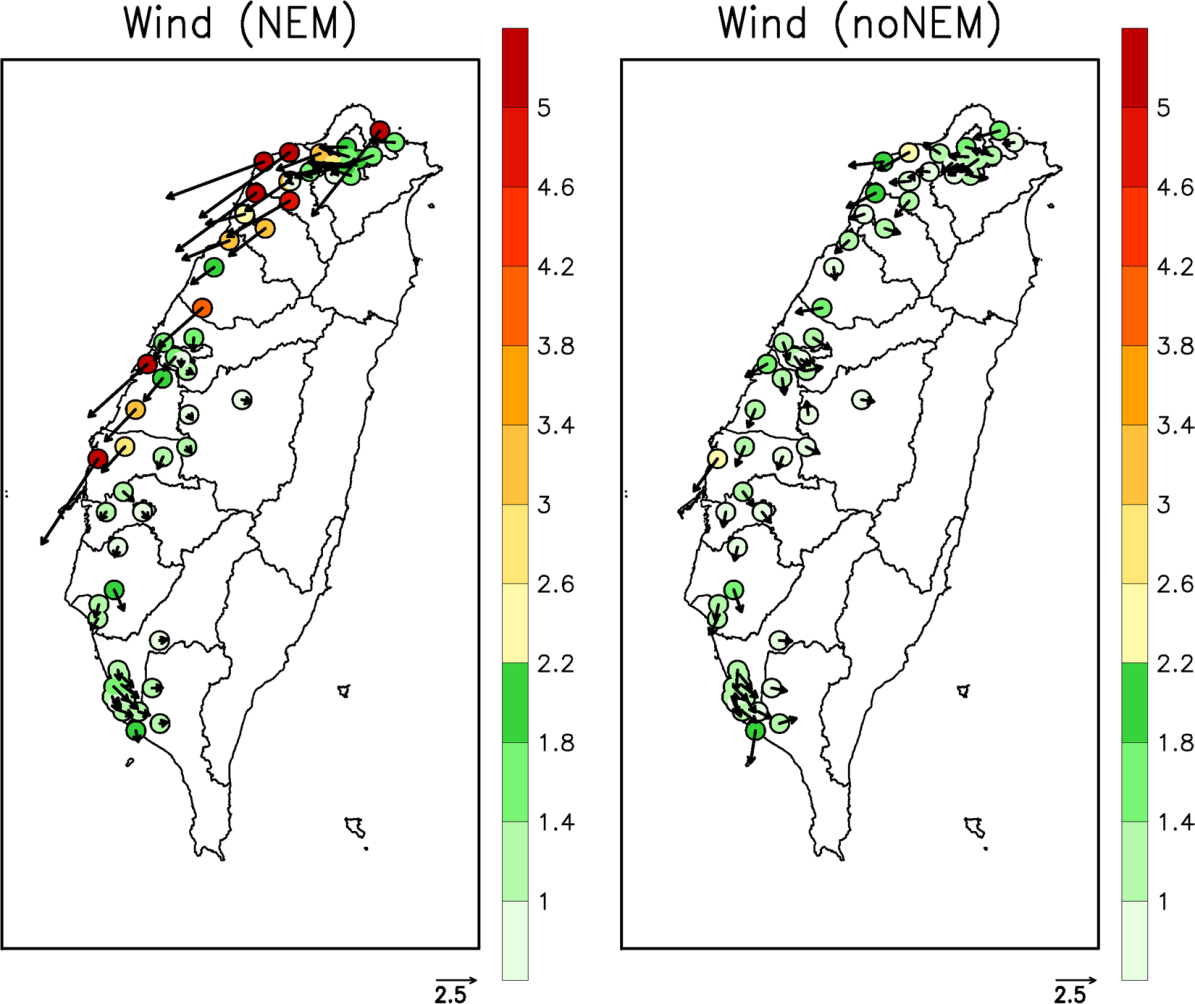
Distribution of the mean wind fields (m s^−1^) at the TEPA surface stations. The data are averaged for the severe PM_2.5_ events from 2007 to 2017. Left and right panels are for the NEM and noNEM cases, respectively.

Depending on the strength of the NE wind flow, the impact of transboundary air pollution on air quality in Taiwan can vary. Generally, northern Taiwan (NT/CM) has high exposure to air pollution from China when the prevailing wind is NE; however, the influence of transboundary air pollution becomes weaker over the CT/YCN/KP areas. Figure 7 presents the distributions of the observed average wind fields during severe PM_2.5_ events (NEM and noNEM) at the individual TEPA surface stations. For the severe PM_2.5_ events of the NEM cases, the NE monsoonal flow prevails in the NT/CM areas and at some coastal stations in the CT/YCN areas. The occurrence of severe PM_2.5_ events for the NEM cases at the inland stations in the CT/YCN areas and in the KP area is associated with weak wind fields, which indicates less influence of transboundary air pollution over those areas. For the severe PM_2.5_ events of the noNEM cases, the wind speeds are generally weaker than those of the NEM cases.

**Figure 8:**
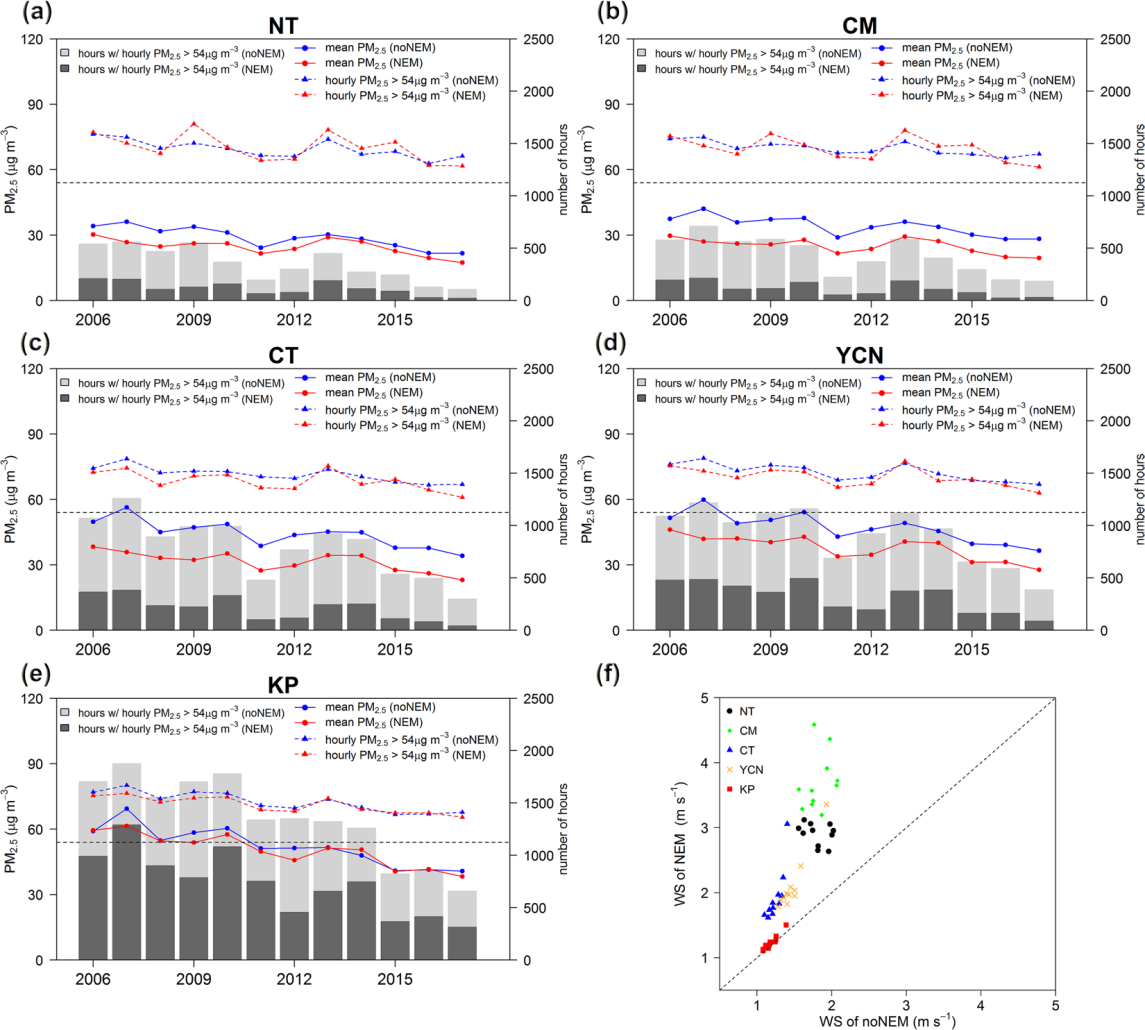
(**a**–**e**) Long-term variations in the PM_2.5_ concentrations. The horizontal black dashed line identifies the PM_2.5_ concentration at 54 μg m^−3^. Blue/red solid lines denote the mean PM_2.5_ concentration of noNEM/NEM cases. Blue/red dashed lines denote the means of the hourly PM_2.5_ concentration >54 μg m^−3^ for the noNEM/NEM cases. Light/dark gray bars indicate the number of hours with an hourly PM_2.5_ concentration >54 μg m^−3^ for the noNEM/NEM cases. (**f**) Annual wind speed comparison between noNEM and NEM cases of severe PM_2.5_ events.

Figure 8 compares the long-term variation in the mean and high PM_2.5_ concentrations and shows an estimate of the number of hours with PM_2.5_ concentrations >54 µg m^−3^ for both NEM and noNEM cases. Figure 8 also displays an annual wind speed comparison using the TEPA surface stations between the NEM and noNEM cases of severe PM_2.5_ events. The mean PM_2.5_ concentrations of the NEM cases are generally lower than those of the noNEM cases. In the NT/CM areas, there has been an apparent reduction in the PM_2.5_ concentrations of the NEM cases since 2013, and the occurrence frequency of severe PM_2.5_ events in the NEM cases has been reduced to a very low number during the recent analysis period, indicating a great improvement in the transboundary air pollution problem. The PM_2.5_ concentrations of the severe PM_2.5_ events are comparable between the NEM and noNEM cases in the NT/CM areas; in some years (2009 and 2013–2015), the PM_2.5_ concentrations are higher for NEM events than for noNEM events due to several heavy PM_2.5_ episodes in China.

Over the CT/YCN/KP areas, the PM_2.5_ concentrations of the severe PM_2.5_ events are generally higher in the noNEM cases than in the NEM cases, except for 2013 and 2015, during which several heavy haze episodes occurred in China and affected Taiwan^37,38^. The wind speed is generally much weaker over the CT/YCN/KP areas than over the NT/CM areas. In the KP area, the wind conditions are very stagnant under severe PM_2.5_ events for both NEM and noNEM cases. Hsu and Cheng^4^ indicated that the CT/YCN/KP areas tend to exhibit stagnant conditions when under the influence of prevailing NE wind due to their location over the leeside of the mountain. The occurrence of severe PM_2.5_ events in the NEM cases over the CT/YCN areas is possibly attributed to transboundary air pollutants, upstream local air pollutants and surrounding local emissions. In the KP area, it can be concluded that most of the severe PM_2.5_ events are mainly caused by locally released emissions accompanied with stagnant wind conditions, with weak influence by transboundary air pollutants. During the winter season of 2013–2014, with a historically high record for the China haze event, the observed data reveal an apparent increase in the PM_2.5_ concentration in not only the NT/CM areas but also the CT/YCN/KP areas. The PM_2.5_ concentrations of the severe PM_2.5_ events are higher in the NEM cases than in the noNEM cases in all the studied AQZs in 2013.

## Discussion

The gradual decline in the PM_2.5_ concentration indicates that the current emissions control strategy is able to improve the general PM_2.5_ problem in Taiwan and reduce the ambient PM_2.5_ concentration. A significant reduction in the PM_2.5_ concentration can be seen in 2008, 2011 and 2015 (Fig. 3). The reductions in 2008 are due to the global recession^43^. An apparent decrease in the PM_2.5_ concentrations in 2011 may be due to the stronger synoptic wind flow (Fig. 6), together with the application of a stricter regulation limiting the sulfur content in diesel and gasoline oil and the subsidy of the electric vehicle battery exchange system^2,44^ in Taiwan. All the AQZs reveal a significant decrease in the PM_2.5_ exceedance days, except in the KP area in 2011 due to the PM_2.5_ concentration being far above the standard. The apparent decrease in the number of PM_2.5_ exceedance days in 2015 in all the AQZs can be partially associated with the lessened influence from long-range transported air pollutants (Fig. 8) and the application of the Clean Air Act^2,44^ for improving the PM_2.5_ problems in Taiwan.

Under regional climate change, a decreasing trend of wind speed and an increasing trend of stagnant wind conditions have occurred over the past 39 years, and a trend of enhanced stably stratified atmospheric structures have been observed in the past decade in Taiwan. Worsening meteorological conditions can limit air pollution dispersion and degrade air quality.

The PM_2.5_ concentrations and the PM_2.5_ exceedance days increase from the northern to southern part of western Taiwan (Fig. 3), and the reductions in the PM_2.5_ concentration generally decrease from northern to southern Taiwan (Table 1). Mitigation of the PM_2.5_ problem is less effective in the KP area than in the other AQZs. Because the KP area is situated on the leeside of the mountain, this area often exhibits stagnant wind conditions when under the influence of NE wind flow^4^. During the six-month winter season of 2017–2018, 67% of the sampling days still violated the daily PM_2.5_ standard, and there were no apparent reductions in the PM_2.5_ concentrations and the PM_2.5_ exceedance days during the most recent three-year period (2015–2017) in the KP area.

Reduction in the PM_2.5_ concentrations in the KP area can also be difficult due to the worsening meteorological conditions. Hou and Wu^17^ also indicated that extreme air pollution meteorology, such as heat waves, temperature inversions and stagnation episodes, has the greatest impact on high levels of air pollution. Finally, we can conclude that the long-term declining trend of PM_2.5_ in Taiwan is mainly associated with changes in local anthropogenic emissions and modulated by short-term yearly variations due to strong haze events in China. In the KP area, the frequent occurrence of stagnant wind fields often leads to high PM_2.5_ concentrations during the air pollution season, and reductions in the PM_2.5_ concentrations are possibly offset by the worsening weather conditions.

In the KP area, improving the PM_2.5_ concentrations has become a challenging task due to the adverse effect of meteorological conditions. To successfully reduce the high PM_2.5_ concentrations, a stringent and effective emission reduction plan needs to be designed in a proper manner by decision-makers. Tasks such as quantifying the contributions of air pollutants by different source sectors and regions is important for designing effective control strategies.

Because climate change and global warming happen worldwide, the findings from this study can be applied in other areas, especially in East Asia, which is also facing similar environmental problems. If this trend continues in the future, stricter emissions reduction plans will be needed by the government of Taiwan to compensate for the adverse effects of worsening meteorological conditions due to regional climate change, particularly in areas that frequently experience stagnant weather conditions.

## Methods

We examined variations in meteorological conditions caused by regional climate change and studied the implications of these variations on surface PM_2.5_ concentrations in Taiwan through a long-term trend analysis of surface observations and global reanalysis datasets. Various trend and annual variation analyses were conducted for NO_x_, SO_2_ and PM_2.5_ concentrations. Sea level pressure and wind field analyses were conducted based on the 39-year ERA-interim reanalysis dataset to examine the long-term variations in the East Asian winter monsoon system. Moreover, the long-term variation in the vertical temperature structure was analyzed to assess the variability in atmospheric stability in Taiwan. The spatial resolution of the dataset is 0.75° latitude by 0.75° longitude, and the temporal resolution of the dataset is 6 hours.

The observed surface NO_x_, SO_2_ and PM_2.5_ concentrations were analyzed using TEPA air quality monitoring stations. The observed NO_x_ and SO_2_ concentrations were available from 1993. The observed PM_2.5_ concentrations were acquired according to the data availability. Currently, 77 air quality monitoring stations are operating in Taiwan. The earliest PM_2.5_ observations started in 1993, although most PM_2.5_ observations were conducted in 2006. The observed surface winds from the 77 TEPA air quality stations were analyzed to present the wind variation in each AQZ. The wind speed comparison presented in Figs 3f and 8f was produced beginning in 2007 according to the data availability. For the long-term wind speed analysis (1979–2017), the CWB surface weather stations, which contain long periods of observations, were used to assess the long-term variations in wind flow in Taiwan. Moreover, the analysis in this study only considers the air pollution season that starts in autumn and lasts until spring; therefore, the data used for the analysis and discussion only include the time period from October to March. The average across the six-month air pollution season is calculated. For example, the average of 2017 is based on data from October 2017 to March 2018, and this method is applied to all of the analyses conducted in this study.

The estimations presented in Table 1 represent the variation ratio of each year relative to the previous year for PM_2.5_ concentrations (PM_2.5_ exceedance days). Then, a mean value is estimated by averaging the variation ratio of each study year (2006–2017).

Instead of presenting a long-term analysis from each station side-by-side, the analysis is conducted based on the AQZ division to develop an integrated understanding of the PM_2.5_ variations in Taiwan. The observed data are averaged from the individual stations located within the AQZs to represent the mean air pollution behavior for each AQZ. Previous studies have mainly targeted certain portions of the Taiwan area^25^ (e.g., northern, central or southern Taiwan) or certain representative stations located in different areas of Taiwan^23^. To our knowledge, this is the first official work conducted in Taiwan that has assessed the long-term PM_2.5_ variations based on the definitions of the AQZs. The advantages include the presentation of long-term PM_2.5_ problems in a comprehensive and integrated manner in different area of Taiwan and the development of a greater understanding of problems associated with emissions and daily changing weather conditions in a particular AQZ.

For the analysis of the severe transboundary PM_2.5_ events, a criterion of 54 µg m^−3^ is chosen based on the definitions of the Air Quality Index (AQI). The TEPA uses the AQI to report daily air quality conditions. The AQI, which is defined according to the United States EPA, is divided into six levels (good, moderate, unhealthy for sensitive groups, unhealthy, very unhealthy, and hazardous). When the AQI changes from unhealthy for sensitive groups to unhealthy level for the general public, the PM_2.5_ concentration crosses 54 µg m^−3^; therefore, PM_2.5_ >54 µg m^−3^ is used to represent severe PM_2.5_ events in this study. However, a daily mean PM_2.5_ >54 µg m^−3^ can be too strict to represent high PM_2.5_ concentrations. For example, the influence of long-range transported air pollution from China can last from a few hours to longer than 24 hours, and a PM_2.5_ event with a shorter duration would be excluded from the discussion of high PM_2.5_ concentration. Thus, an approach considering hourly PM_2.5_ concentrations >54 µg m^−3^ is analyzed to characterize the problems associated with severe PM_2.5_ events.

## Supplementary information

SupplementaryInformation

## Data Availability

The global reanalysis datasets used in this paper are available at https://www.ecmwf.int/en/forecasts/datasets/archive-datasets/reanalysis-datasets/era-interim. The observed surface datasets that support the findings of this study are available from the corresponding author upon reasonable request.

## References

[1] Fang SH, Chen HW (1996). Air quality and pollution control in Taiwan. Atmos. Environ..

[2] Tsai WT (2016). Current status of air toxics management and its strategies for controlling emissions in Taiwan. Toxics.

[3] Yen CH, Horng JJ (2009). Volatile organic compounds (VOCs) emission characteristics and control strategies for a petrochemical industrial area in middle Taiwan. J. Environ. Sci. Health A Tox. Hazard Subst. Environ. Eng..

[4] Hsu, C. H. & Cheng, F. Y. Synoptic weather and associated air pollution patterns in Taiwan. *Aerosol Air Qual. Res*. In press (2019).

[5] Tsai HH, Ti TH, Yuan CS, Hung CH, Lin C (2008). Effects of sea-land breezes on the spatial and temporal distribution of gaseous air pollutants at the coastal region of southern Taiwan. J. Environ. Eng. Manag..

[6] Tsai HH, Yuan CS, Hung CH, Lin C (2011). Physicochemical properties of PM_2.5_ and PM_2.5–10_ at inland and offshore sites over Southeastern coastal region of Taiwan strait. Aerosol Air Qual. Res..

[7] Lai L-W (2014). Relationship between fine particulate matter events with respect to synoptic weather patterns and the implications for circulatory and respiratory disease in Taipei, Taiwan. Int. J. Environ. Health Res..

[8] Cheng FY, Chin SC, Liu TH (2012). The role of boundary layer schemes in meteorological and air quality simulations of the Taiwan area. Atmos. Environ..

[9] Tsai DM, Wu YL (2006). Effects of highway networks on ambient ozone concentrations - a case study in Southern Taiwan. Atmos. Environ..

[10] Chuang MT (2008). Simulation of long-range transport aerosols from the Asian continent to Taiwan by a south- ward Asian high-pressure system. Sci. Total Environ..

[11] Fang GC (2017). Seasonal variations and sources study by way of back trajectories and ANOVA for ambient air pollutants (particulates and metallic elements) within a mixed area at Longjing, central Taiwan: 1-year observation. Environ. Geochem. Health.

[12] Garcia-Menendez F, Monier E, Selin NE (2017). The role of natural variability in projections of climate change impacts on U.S. ozone pollution. Geophys. Res. Lett..

[13] Gidhagen L, Engardt M, Lövenheim B, Johansson C (2012). Modeling effects of climate change on air quality and population exposure in urban planning scenarios. Adv. Meteorol..

[14] Leung LR, Gustafson WI (2005). Potential regional climate change and implications to US air quality. Geophys. Res. Lett..

[15] Pommier M (2018). Impact of regional climate change and future emission scenarios on surface O_3_ and PM_2.5_ over India. Atmos. Chem. Phys..

[16] Cheng FY, Jian SP, Yang ZM, Tsuang BJ, Yen MC (2015). Impact of regional climate changes on meteorological characteristics and their subsequent effect on ozone dispersion in Taiwan. Atmos. Environ..

[17] Hou P, Wu S (2016). Long-term changes in extreme air pollution meteorology and the implications for air quality. Sci. Rep..

[18] Fiddes SL, Pezza AB, Mitchell TA, Kozyniak K, Mills D (2016). Synoptic weather evolution and climate drivers associated with winter air pollution in New Zealand. Atmos. Pollut. Res..

[19] Mu Q, Liao H (2014). Simulation of the interannual variations of aerosols in China: role of variations in meteorological parameters. Atmos. Chem. Phys..

[20] Kim HC (2017). Recent increase of surface particulate matter concentrations in the Seoul metropolitan area, Korea. Sci. Rep..

[21] Hsu HH, Chen CT (2002). Observed and projected climate change in Taiwan. Meteorol. Atmospheric Phys..

[22] Shiu CJ, Liu SC, Chen JP (2009). Diurnally asymmetric trends of temperature, humidity, and precipitation in Taiwan. J. Clim..

[23] Lee CS, Chang KH, Kim H (2018). Long-term (2005–2015) trend analysis of PM_2.5_ precursor gas NO_2_ and SO_2_ concentrations in Taiwan. Environ. Sci. Pollut. Res. Int..

[24] Chen SP (2014). Recent improvement in air quality as evidenced by the island-wide monitoring network in Taiwan. Atmos. Environ..

[25] Chang SC, Lee CT (2007). Evaluation of the trend of air quality in Taipei, Taiwan from 1994 to 2003. Environ. Monit. Assess..

[26] Jung CR, Hwang BF, Chen WT (2017). Incorporating long-term satellite-based aerosol optical depth, localized land use data, and meteorological variables to estimate PM_2.5_ concentrations in Taiwan from 2005 to 2015. Environ. Pollut..

[27] Dee DP (2011). The ERA‐interim reanalysis: configuration and performance of the data assimilation system. Q. J. R. Meteorol. Soc..

[28] Wang H, He S (2011). Weakening relationship between East Asian winter monsoon and ENSO after mid-1970s. Chin. Sci. Bull..

[29] Sherwood SC, Nishant N (2015). Atmospheric changes through 2012 as shown by iteratively homogenised radiosonde temperature and wind data (IUKv2). Env. Res. Lett..

[30] Horton DE, Skinner CB, Singh D, Diffenbaugh NS (2014). Occurrence and persistence of future atmospheric stagnation events. Nat. Clim. Chang..

[31] Liu J, Gao Z, Wang L, Li Y, Gao CY (2017). The impact of urbanization on wind speed and surface aerodynamic characteristics in Beijing during 1991–2011. Meteorology and Atmospheric Physics.

[32] Lopes A, Saraiva J, Alcoforado MJ (2011). Urban boundary layer wind speed reduction in summer due to urban growth and environmental consequences in Lisbon. Environ. Model Softw..

[33] Zhang X, Zhong J, Wang J, Wang Y, Liu Y (2018). The interdecadal worsening of weather conditions affecting aerosol pollution in the Beijing area in relation to climate warming. Atmos. Chem. Phys..

[34] Chen L, Li D, Pryor SC (2013). Wind speed trends over China: quantifying the magnitude and assessing causality. Int. J. Climatol..

[35] Fu G (2011). Temporal variation of wind speed in China for 1961–2007. Theor. Appl. Climatol..

[36] Niu F, Li Z, Li C, Lee K-H, Wang M (2010). Increase of wintertime fog in China: potential impacts of weakening of the Eastern Asian monsoon circulation and increasing aerosol loading. J. Geophys. Res. Atmos..

[37] Wang SH, Hung WT, Chang SC, Yen MC (2016). Transport characteristics of Chinese haze over Northern Taiwan in winter, 2005–2014. Atmos. Environ..

[38] Chuang MT, Lee CT, Hsu HC (2018). Quantifying PM_2.5_ from long-range transport and local pollution in Taiwan during winter monsoon: an efficient estimation method. J. Environ. Manage..

[39] Zhang Z, Ma Z, Kim SJ (2018). Significant decrease of PM_2.5_ in Beijing based on long-term records and Kolmogorov-Zurbenko filter approach. Aerosol Air Qual. Res..

[40] Wang X, Dickinson RE, Su L, Zhou C, Wang K (2017). PM_2.5_ pollution in China and how it has been exacerbated by terrain and meteorological conditions. Bull. Am. Meteorol. Soc..

[41] Zheng B (2018). Trends in China’s anthropogenic emissions since 2010 as the consequence of clean air actions. Atmos. Chem. Phys..

[42] Junker C, Wang J-L, Lee C-T (2009). Evaluation of the effect of long-range transport of air pollutants on coastal atmospheric monitoring sites in and around Taiwan. Atmos. Environ..

[43] Castellanos P, Boersma KF (2012). Reductions in nitrogen oxides over Europe driven by environmental policy and economic recession. Sci. Rep..

[44] Environmental Protection Administration, Executive Yuan, R.O.C., Taiwan. Documentary on air quality protection, https://www.epa.gov.tw/eng/5FF11AF44EF9533B (2017).

