# Supplementary material for: Long-term variations in PM2.5 concentrations under changing meteorological conditions in Taiwan
Source: Sci Rep. 2019 Apr 29;9:6635. doi: 10.1038/s41598-019-43104-x (PMC6488571; doi:10.1038/s41598-019-43104-x)
Supplement: Supplementary file 1 — SupplementaryInformation [file 41598_2019_43104_MOESM1_ESM.pdf]

Supporting Information

Long-term variations in $PM_{2.5}$ concentrations under changing meteorological conditions in Taiwan

Fang-Yi Cheng and Chia-Hua Hsu

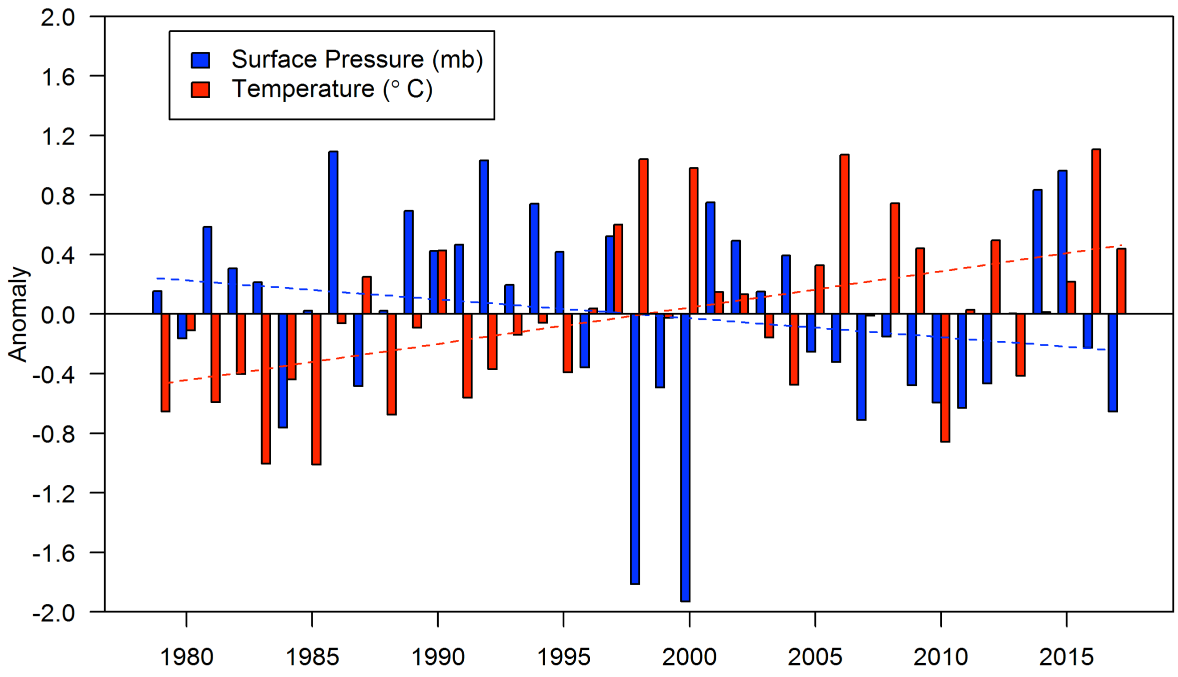

Fig. S1. Long-term variation (1979–2017) of the observed surface temperature and pressure averaged from the surface CWB weather stations in Taiwan.

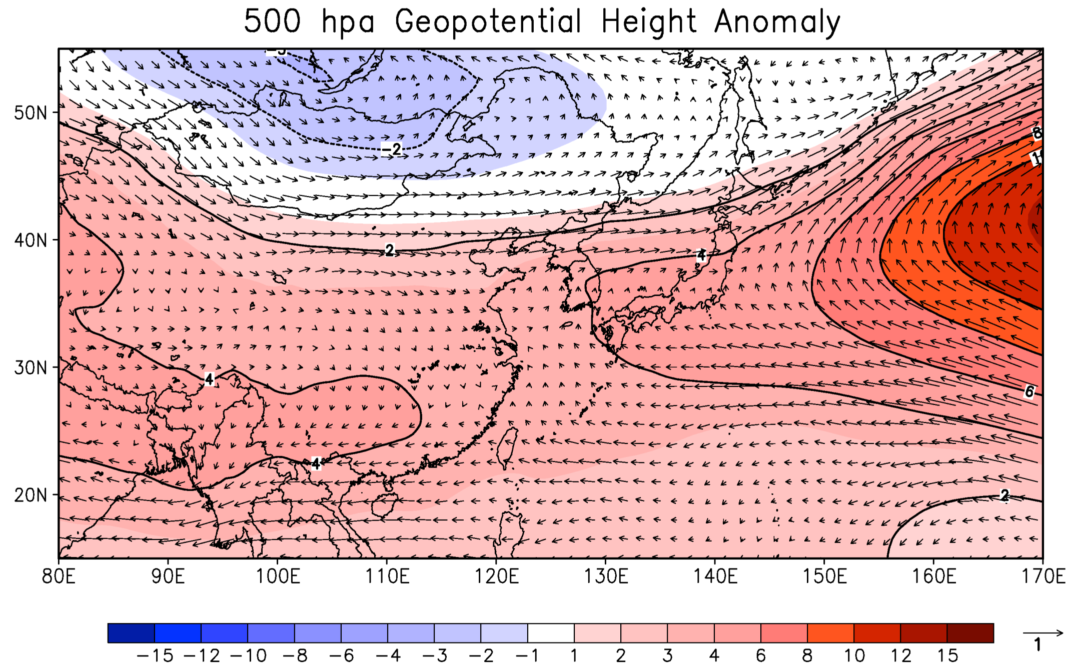

Fig. S2. Distribution of the 500-hPa geopotential height anomaly (m). Shaded colors and wind vectors represent the anomaly of the geopotential height and wind (m s^{-1}) from 1997 to 2017 relative to the 39-year (1979 to 2017) average.
